# Supplementary material for: Are carbon emissions trading and green financial instruments synergistic? -Comprehensive quantitative research based on content analysis
Source: PLoS One. 2024 Mar 7;19(3):e0298601. doi: 10.1371/journal.pone.0298601 (PMC10919601; doi:10.1371/journal.pone.0298601)
Supplement: S1 Data — (ZIP) [file pone.0298601.s001.zip › Data/地级市Z_F工作报告.zip!/╜¡╦╒/╬▐╬2╩╨/└┤╫╘╣1⁄2╓┌║┼í╛┬φ┐╦╩2╛▌═°í┐_2018.docx]

各位代表：

　　现在，我代表无锡市人民政府，向大会报告政府工作，请予审议，并请市政协委员和其他列席人员提出意见。

　　一、2017年工作回顾

　　2017年是党的十九大胜利召开之年，也是全面落实省、市第十三次党代会决策部署的开局之年和本届政府依法履职的第一年。面对复杂多变的宏观经济形势，在中共无锡市委的坚强领导下，在市人大、市政协的监督和支持下，市政府认真学习宣传贯彻党的十九大精神，以习近平新时代中国特色社会主义思想为指导，紧紧依靠全市广大人民，牢牢把握稳中求进工作总基调，坚定不移贯彻新发展理念，深入开展“两聚一高”新实践，大力实施六大发展战略，全力推进“强富美高”新无锡建设，圆满完成市委十三届二次全会和市十六届人大一次会议确定的目标任务，为无锡新时代实现新跨越打下了坚实基础。

　　（一）综合实力迈上新的台阶。预计全市实现地区生产总值10500亿元，同比增长7.4%，增速五年来首次超过全省平均水平，这标志着无锡经济发展实现了历史性跨越、城市能级和综合实力显著增强。预计规模以上工业增加值达3330亿元，增长8.5%，增速创近五年新高。社会消费品零售总额达3460亿元，增长11%，增速十年来首次超过全省平均水平。固定资产投资达4915亿元，增长4.6%。外贸进出口超过800亿美元，增长15%。一般公共预算收入930亿元，增长6.3%。全社会研发投入占地区生产总值比重提高到2.86%，科技进步贡献率提高到63.5%，保持全省领先水平。12家企业入围中国企业500强，位居全省第一；24家企业入围中国制造业500强，占全省半数以上。江阴、宜兴分列中国工业百强县（市）第一位和第八位。海澜集团成为全市首家营业收入超千亿元的企业。社会融资规模达1650亿元，增长11.5%；金融机构本外币存、贷款余额分别达15123亿元和11233亿元，增长3.5%和6.8%，其中制造业贷款占比27.7%、全省最高。新增境内外上市企业18家，累计达129家，上市公司总量位居全省第一。

　　（二）产业强市建设成效明显。重大项目建设取得新突破。列入年度计划的100个市级重大项目全部开工建设，当年完成投资705.8亿元。全市新签约重大产业项目44个，计划总投资3910亿元，其中300亿元以上项目3个、100亿元以上项目10个，单体规模刷新历史纪录。突出发展具有比较优势的战略性新兴产业。制定实施《加快发展以物联网为龙头的新一代信息技术产业三年行动计划》，成功举办2017世界物联网博览会，华为、阿里巴巴、腾讯、中电海康等一大批高科技企业项目相继落户，鸿山物联网小镇建设顺利推进，雪浪、慧海湾物联网小镇和浪潮大数据产业园、国家智能交通综合测试基地启动建设，物联网产业营业收入增长18%，无锡作为物联网发轫地的影响力和辐射力不断提升。华虹集成电路研发和制造、天津中环大硅片研发和制造、SK海力士第二工厂等行业龙头项目相继落地，江阴高新区获批国家集成电路封测高新技术产业化基地，无锡作为集成电路重要产业基地的地位得到巩固和提升。突出发展具有领先优势的智能制造。制定实施《智能制造三年行动计划》，积极开展“中国制造2025”苏南城市群试点示范，探索军民融合发展无锡路径，组织实施270个智能化改造项目，新增省级示范智能车间17家，惠山智能制造物流装备产业基地获批国家火炬特色产业基地，阳光集团成为全省首家荣获中国质量奖的企业，兴澄特钢获得中国质量奖提名奖，法尔胜泓昇集团获得2017年江苏省省长质量奖。突出发展具有特色优势的现代服务业。制定实施《现代服务业提质增效三年行动计划》，预计服务业增加值占地区生产总值比重提高到51.5%。成功入选首批十大“中国旅游休闲示范城市”和体育旅游十佳目的地，预计旅游总收入和接待游园人数分别增长10.2%、6.8%，灵山禅意小镇、湖氵父茶旅小镇、阳山桃源小镇入选全省首批旅游风情小镇。进一步完善会展经济政策，全年举办各类会展活动130个，获得“中国十佳品牌会展城市”称号。加快发展现代农业。新增高标准农田1万亩、高效设施农业（渔业）1.5万亩，无锡国家现代农业示范区和农业基本现代化综合得分继续保持全省全国前列，锡山现代农业产业园获批创建国家现代农业产业园，东港镇红豆杉康养小镇等8个小镇入选省农业特色小镇培育计划。完成第三次全国农业普查任务。

　　（三）科技创新能力不断增强。区域创新竞争力持续提高。围绕苏南国家自主创新示范区建设目标，组织实施创新驱动核心战略30条、知识产权强市建设15条等政策措施，科技进步综合评价排名上升到全省第二位。4项科技成果获国家科学技术奖，“神威·太湖之光”超级计算机实现全球超算500强榜单四连冠、应用成果再获戈登·贝尔奖。8个项目获第十九届中国专利奖，万人有效发明专利拥有量达34件。智慧城市建设持续推进，在全国智慧城市发展水平评选中连续五年名列前茅。企业创新主体地位持续提升。新增省级工程技术研究中心22家、省级企业技术中心23家、国家技术创新示范企业1家，预计企业研发经费占销售收入比重提高到1.75%，列全省第一。深入实施高新技术企业培育计划，新增省级科技小巨人企业4家，全市高新技术企业总数达1669家，高新技术产业产值达6670亿元、增长18%。实施“强内拓外”产学研工程，推动企业与140多家高校院所建立校企联盟910家，推进建设产学研合作新型研发机构21家、院士工作站139家。人才引进培养力度持续加大。实施“太湖人才计划”升级版，举办高层次人才创新创业无锡交流大会、“百企千才高校行”和青年企业家基业长青“百千万工程”活动，全年新引进各类人才7.5万人，江阴高新区诺贝尔奖得主设立研究院数量居全省开发区首位，省级众创空间、创业示范基地分别达到36家、24家。

　　（四）改革开放水平全面提升。供给侧结构性改革深入推进。化解钢铁产能90万吨，市区非住宅商品房库存减少19.35万平方米，政府性债务规模实现管控目标，全年继续为企业减负超过270亿元，启动运营“锡信贷”业务，为中小企业提供转贷应急资金超过100亿元，银行业金融机构不良贷款余额和不良贷款率连续四年实现“双下降”，不良贷款率下降到1.20%。“放管服”改革扎实推进。开展相对集中行政许可权改革试点，市行政审批局挂牌成立，市级继续向市（县）、区下放行政权力事项86项，省委省政府确定的江阴市县级集成改革试点全面启动，徐霞客镇经济发达镇行政管理体制改革试点经验在全国推广，宜兴官林镇等3个镇入选全省新一轮经济发达镇行政管理体制改革名单。全面推行“不见面”审批（服务），向社会公布“不见面”审批（服务）事项清单167项，有效落实省定“3550”改革目标。商事制度改革成效显著，“多证合一、一照一码”改革覆盖范围不断扩大，“双随机、一公开”抽查工作全面展开，全市新登记内资企业数量和注册资本金额分别增长21.5%、13.5%。重点领域改革协同推进。国有企业改革迈出重要步伐，成立市属国有资本运营公司，推进市属城建类国有企业合并重组，发展混合所有制项目55个，吸收社会资本投资341亿元。优化完善市对区财政体制，扩大信保基金规模和覆盖面，9个PPP项目落地实施，国税地税征管体制改革取得阶段性成效。农村土地确权颁证基本完成，农村产权交易市场基本组建到位。承担行政职能的事业单位改革试点基本完成。医药卫生体制改革不断深化，分级诊疗体系加快构建。全国养老服务综合改革试点取得实效，养老服务品牌化、连锁化、社会化发展势头良好。

　　开放型经济稳中向好。“引进来”“走出去”取得新成绩。外贸发展方式加快转变，79家企业入围省级出口品牌，预计外贸出口总额突破490亿美元，增长13%。制定扩大利用外资政策意见，完成到位注册外资36.7亿美元，其中制造业占比60%，新增跨国公司地区总部和功能性机构7家。完成境外投资中方协议投资额12亿美元，柬埔寨西港特区入驻企业达到118家，无锡一棉埃塞俄比亚纺织生产基地项目正式签约。开放口岸和平台建设取得新进展。进口肉类指定口岸建成投运，苏南硕放国际机场旅客吞吐量668万人次、增长20.2%，货邮吞吐量10.75万吨、增长12.1%，无锡（江阴）港货物吞吐量首次突破2亿吨。成功举办江苏发展大会无锡乡贤恳谈会、第三届全球锡商大会、2017无锡—香港智能制造和现代服务业合作恳谈会、徐霞客诞辰430周年纪念大会暨第十二届徐霞客国际旅游节、中日樱花友谊林建设和《无锡旅情》唱响30周年纪念活动等，城市开放度、知名度进一步提高。

　　（五）城乡建设发展步伐加快。城市规划和空间布局持续优化。新一轮城市总体规划形成中期成果，梁溪区、新吴区行政区划调整成效逐步放大，太湖新城、锡东新城、惠山新城产业布局和配套功能进一步完善，历史城区“双修”试点启动。基础设施建设加速推进。制定锡澄锡宜重大基础设施分年度建设计划，苏锡常南部高速公路无锡段、常宜高速公路开工建设，宜长高速公路先导段启动建设，锡澄运河市区段航道整治工程加快实施。锡澄城际轨道S1线前期工作取得阶段性进展，地铁1号线南延线提前贯通，地铁3号线一期完成60%的隧道掘进量，地铁4号线一期10座车站进入主体施工，地铁1号线荣获国家优质工程“金奖”。蠡湖大道快速化改造高架段、广益路、吴桥西路等重点道桥项目建成通车，江海西路快速化改造完成50%工作量。江阴窑港水源地取水口迁建工程完成，城乡区域供水覆盖率达100%。老城区更新改造步伐加快。市区完成65万平方米棚户区和217万平方米旧住宅区整治改造任务。拆除违法建设77万平方米，完成26条主要道路包装出新和116个背街小巷综合治理，被授予“省优秀管理城市”称号。新增、改造居民天然气用户7万户。地铁1、2号线日均客流量同比增加11.4%，新辟优化公交线路31条，新增低碳环保公交车170辆，公共自行车一期项目建成投运。广南立交绿地改造等一批海绵城市建设重点项目有序推进，无锡入选省级海绵城市建设试点。新农村建设稳步推进。出台《关于加强和改进农村住房建设管理的意见（试行）》，支持符合条件的农村居民翻建或新建住房。因地制宜建设美丽乡村，江阴新桥镇等3个镇获得第二批全国特色小镇称号，江阴红豆村获评中国美丽休闲乡村，宜兴张阳村获评全国美丽乡村示范村，江阴璜土村获评全国改善农村人居环境示范村，惠山区阳山镇冯巷等3个村入选江苏省首批特色田园乡村建设试点。

　　（六）生态环境质量持续改善。环境综合整治取得新进步。扎实开展“263”专项行动，大力推进中央、省环保督察和环保部太湖治理专项督导反馈问题整改，成功跻身首批国家生态文明建设示范市。全面开展新一轮太湖治理和河道综合整治，完成九里河、伯渎港等区域性骨干河道和市区10条黑臭河道整治任务，新建、改建污水管网114公里，规模养殖场治理率达到70%，新孟河水利工程启动建设，加大蓝藻和水草打捞处置力度，45个国考省考断面达到或优于III类水质比例同比提高20个百分点，太湖无锡水域水质总体向好，连续第十年实现安全度夏。完成大气污染防治10大类267项重点工程，空气质量优良天数比例达到67.7%，PM2.5平均浓度同比下降14.6%。制定实施《市区主要固体废弃物处理设施建设三年计划》，生活垃圾分类收集全面推广，锡东垃圾焚烧发电厂建成投运，生活垃圾无害化处理率保持100%。节约集约和绿色发展取得新成效。市县乡三级土地利用总体规划调整方案获批，全市划定永久基本农田137.13万亩，工业企业资源利用绩效评价工作全面启动，单位建设用地GDP产出预计达6.93亿元/平方公里。万元GDP能耗、水耗进一步下降，化学需氧量、氨氮、二氧化硫、氮氧化物四项主要污染物排放量削减超额完成考核任务。整治燃煤锅炉28台、燃煤工业窑炉141座，减少煤炭消耗总量109万吨，淘汰低端落后化工企业275家。新增城市绿地面积205万平方米，全市林木覆盖率达到27%，显义桥绿地等一批城市游园建成开放。宛山荡湿地公园成为省级湿地公园，全市自然湿地保护率达到50%，率先在省内建成全市域湿地群。加强生态文明制度建设。出台生态环境保护工作责任规定，调整完善生态补偿政策，积极推进水环境区域补偿和生态重点区域补偿工作，继续开展排污权有偿使用和交易以及环境污染责任保险试点，为生态文明建设提供了有力保障。

　　（七）人民生活水平稳步提高。民生福祉持续改善。突出聚焦富民的发展导向，组织实施聚焦富民33条政策措施，财政资金用于民生的比例达80%左右，为民办实事10大项目全面完成。新增城镇就业15.2万人，扶持自主创业1.3万人，全体居民人均可支配收入增长8.5%左右。基本养老、医疗、失业、工伤和生育保险分别扩面6.37万人、6.01万人、5.87万人、3.53万人、3.53万人。企业退休人员养老金、居民基础养老金和市区城镇低保标准分别提高到每人每月2649元、405元和820元。市区大病保险受惠群众累计达20万人次。增设养老床位2000张，新建街道（镇）级日间照料中心14家。建成公租房1100套，新增审批通过住房保障家庭2000户。实现全市经济薄弱村脱困转化40个。社会事业加快发展。开工建设幼儿园和中小学新建、改扩建项目79个，4所学校建成省现代化示范性（优质特色）职业学校，新少年宫建成。启动与江南大学新一轮合作共建，江南大学两个学科入选“双一流”建设学科，太湖学院入选省硕士学位授予立项建设单位，南京信息工程大学滨江学院无锡校区开工建设，江南影视艺术职业学院“专升本”列入省“十三五”规划。第五人民医院新院区建成投用，第四人民医院新院区主体工程封顶，27家社区卫生服务机构提档升级，医联体总数达到18家，普仁医疗集团成立运行。传统锡剧《珍珠塔》、民族歌剧《二泉》、舞剧《画女情怀》入选国家重点资助扶持剧目，惠山古镇加入“江南水乡古镇”联合申遗。城乡社区“10分钟体育健身圈”实现全覆盖，成功举办亚洲乒乓球锦标赛、世界跆拳道大满贯冠军系列赛、第十二届市运会等重大体育赛事。社会治理成效显著。江阴、宜兴双双入选全国文明城市，无锡率先创建成为全国文明城市群。全市96%以上的社区达到省级和谐社区标准。安全生产形势总体稳定，连续16年实现事故起数、死亡人数“双下降”。切实加强食品药品安全监管，江阴、宜兴创建成为省级食品安全示范市。扎实推进平安无锡、法治无锡建设，群众安全感、法治建设满意度均列全省第一，连续三届荣获“全国社会治安综合治理优秀市”称号，被授予最高奖“长安杯”。史志、档案、外事、港澳、对台、侨务、民族宗教、人武、人防、双拥、气象、防震减灾等工作取得新成绩，妇女、儿童、老龄、残疾人、红十字等事业有了新发展。

　　（八）政府自身建设进一步加强。深入学习宣传贯彻党的十九大精神，切实增强“四个意识”，坚定在政治上思想上行动上同以习近平同志为核心的党中央保持高度一致，扎实推进“两学一做”学习教育常态化制度化。全面推进依法行政，提请市人大常委会审议地方性法规2件，制定出台市政府规章4件，集中开展政府规章规范性文件清理，完成重大事项合法性审查62件。自觉接受人大工作监督、法律监督和政协民主监督，深入执行市人大及其常委会的决议和决定，定期向市人大常委会报告重大事项和重要工作，认真听取市政协和各民主党派、工商联、无党派人士、人民团体意见，按时办复247件市人大代表议案建议和341件市政协委员提案，代表和委员满意率分别达到97.6%和98.4%。改进政府工作作风，深入开展“连心富民、联企强市”大走访活动，有效解决企业和群众反映的各类问题11.84万件。大力推进政务公开，滨湖区列为全国基层政务公开标准化规范化试点单位，中国无锡政府门户网站实现全国地市级政府网站绩效评估七连冠。认真执行中央八项规定和省、市委十项规定，市政府“三公”经费支出进一步下降。加强审计监督，完成审计项目358个，整改问题资金64.92亿元。切实加强党风廉政建设，认真抓好巡视巡察反馈问题整改，推进“阳光扶贫”监管系统建设，着力解决发生在群众身边的不正之风和腐败问题，定期通报曝光典型案例，严肃查处一批违纪违法案件。

　　各位代表，这些成绩的取得，是中共无锡市委正确领导的结果，是市人大、市政协有效监督和大力支持的结果，是全市广大干部群众团结一心、努力奋斗的结果。在此，我代表无锡市人民政府，向全市人民，向辛勤工作在乡村、社区、企业等基层组织和单位的广大干部员工，向人大代表、政协委员，向各民主党派、工商联、各人民团体和各界人士，向中央及省在锡单位，向驻锡人民解放军、武警官兵和公安干警，向所有参与、支持和关心无锡建设与发展的海内外朋友，表示衷心的感谢并致以崇高的敬意！

　　同时，我们也清醒地认识到，我市经济社会发展还存在不少困难和问题，政府工作也存在一些差距和不足。主要是：发展质量和效益还不高，实体企业生产经营仍然面临不少困难；　科技创新能力不够强，高新技术产业发展相对滞后，人才引育还不能全面适应转型发展需要；资源环境约束趋紧，节约资源和保护环境任务艰巨；深化改革进展不够平衡，一些改革举措和重点任务需要进一步落实；基础设施、民生改善、社会事业、社会治理等领域还存在不少短板和薄弱环节；有的地区中小企业“互保链”风险和政府性债务风险需要进一步化解；少数政府部门和工作人员担当意识不强、工作效率不高，“庸懒散”和不作为、慢作为现象还不同程度存在，等等。对此，我们将进一步增强忧患意识，直面问题，勇于担当，在今后工作中认真加以解决。

　　二、深入学习贯彻党的十九大精神

　　学习贯彻党的十九大精神，是当前和今后一个时期的首要政治任务。我们要深入学习、认真领会、准确把握党的十九大提出的重要思想、重要论断、重大战略部署、重大政策举措，紧密联系无锡实际抓好贯彻落实，推动“强富美高”新无锡建设取得更大成效。

　　坚持以习近平新时代中国特色社会主义思想为指导。把学习习近平新时代中国特色社会主义思想作为理论武装的核心任务，深入学习领会习近平新时代中国特色社会主义思想的核心要义和丰富内涵，牢固树立“四个意识”，坚决维护以习近平同志为核心的党中央权威和集中统一领导，自觉把习近平新时代中国特色社会主义思想作为建设“强富美高”新无锡的根本遵循，不折不扣把中央和省、市委决策部署贯彻好、落实好。

　　深入贯彻新发展理念、建设现代化经济体系。牢牢把握我国经济由高速增长阶段转向高质量发展阶段的深刻变化，坚持质量第一、效益优先，把质量作为产业强市之基，着力加以巩固和提升。深化供给侧结构性改革，推动经济发展质量变革、效率变革、动力变革。高水平建设国家创新型城市，倡导创新文化，强化人才激励，支持企业创新，加强知识产权保护，加速科技成果转化。实施乡村振兴战略，加快推进农业农村现代化。着力推动区域协调发展。坚持把发展经济的着力点放在实体经济上，加快建设实体经济、科技创新、现代金融、人力资源协同发展的产业体系，努力打造国内一流、具有国际影响的现代产业新高地。

　　更好满足人民日益增长的美好生活需要。适应我国社会主要矛盾变化，坚持以人民为中心的发展思想，把满足人民群众对美好生活需要、着力解决不平衡不充分的发展问题，作为政府工作的努力方向。紧紧抓住人民群众最关心最直接最现实的利益问题，既尽力而为，又量力而行，一件事情接着一件事情办，一年接着一年干，使人民群众有更多的获得感、幸福感、安全感，努力创造民生幸福、百姓充分认可的全面小康新生活。

　　加大生态文明建设力度。增强尊重自然、顺应自然、保护自然意识，坚持节约优先、保护优先、自然恢复为主的方针，加快形成节约资源和保护环境的空间格局、产业结构、生产方式、生活方式。着力解决环境突出问题，深入实施“263”专项行动，切实把太湖治理摆上更加重要位置，着力推进绿色发展，加强生态系统保护，努力建造生态宜人、内涵品质跃升的美丽城乡新家园。

　　大力推动社会主义文化繁荣兴盛。坚持文化自信，弘扬时代主旋律，大力培育和践行社会主义核心价值观，深化公民思想道德建设，传承优秀传统文化，繁荣文艺创作，加快文化事业发展，促进文化产业做强做大，推动全国文明城市创建工作向更高水平迈进，努力塑造体现传承、彰显时代精神的社会文明新气象。

　　切实把全面从严治党要求落实到政府工作之中。推动全市政府系统各级党组织认真履行全面从严治党主体责任，不断加强党的建设，旗帜鲜明讲政治。深入推进“两学一做”学习教育常态化制度化，扎实开展“不忘初心、牢记使命”主题教育。持之以恒正风肃纪，强化监督执纪问责，深入开展反腐败斗争，建立健全对行政权力运行的监督和制约机制。

　　奋力在新时代展现新气象新作为。中国特色社会主义进入新时代，新时代要有新气象、新作为。我们要切实增强责任感、使命感、紧迫感和担当精神，勇于解放思想，善于抢抓机遇，强化创新引领，注重系统提升，坚持底线思维，以功成不必在我的胸怀、低调务实不张扬的作风、撸起袖子加油干的劲头，锐意进取、埋头苦干、不懈奋斗，努力开创政府工作新局面。

　　三、2018年主要工作

　　今年是全面贯彻落实党的十九大精神的开局之年，是改革开放40周年，是决胜高水平全面建成小康社会、实施“十三五”规划承上启下的关键一年，做好今年各项工作意义重大。经过多年的发展和积累，特别是通过近年来奋力攻坚，无锡去年跨上地区生产总值万亿元台阶、跻身首批国家生态文明建设示范市、创成首个全国文明城市群、荣获全国社会治安综合治理“长安杯”，标志着无锡发展站上了新的更高能级和起点。适应新时代要求，谋划好、推动好新的更高能级和起点上的无锡发展，实现高质量发展、总体协调发展，是政府工作必须回答的重大课题和必须扛起的重大责任。

　　今年政府工作的总体要求是：深入学习贯彻党的十九大精神，以习近平新时代中国特色社会主义思想为指导，认真学习贯彻习近平新时代中国特色社会主义经济思想，认真贯彻落实去年中央经济工作会议精神和省委十三届三次全会、市委十三届五次全会精神，坚持稳中求进工作总基调，自觉践行新发展理念，统筹推进“五位一体”总体布局和协调推进“四个全面”战略布局，以高质量发展为导向，以推进供给侧结构性改革为主线，着力推动质量、效率、动力三大变革，坚定实施六大发展战略，聚力创新、聚焦富民，全面做好稳增长、促改革、调结构、惠民生、防风险各项工作，确保经济平稳健康发展，确保人民生活持续改善，确保社会大局和谐稳定，推动高水平全面建成小康社会和建设“强富美高”新无锡取得更大成果。

　　全市经济社会发展主要目标是：地区生产总值增长7—7.5%；一般公共预算收入增长6.5%左右；固定资产投资增长5%左右；社会消费品零售总额增长10%以上；外贸进出口增长5%左右；全社会研发投入占地区生产总值比重达到2.92%；城乡居民收入增长与经济增长同步，城镇登记失业率、居民消费价格涨幅控制在省定范围内；节能减排和大气、水环境质量确保完成省下达的目标任务。确定以上主要目标，是以党的十九大精神为根本遵循，按照中央经济工作会议和省委十三届三次全会、市委十三届五次全会精神，既充分考虑了新时代高质量发展要求，又充分考虑了无锡的基础条件、增长可能和就业需要，为调结构转方式优生态留有空间；既体现了高水平全面建成小康社会的导向，也体现了实事求是和奋发有为的要求。

　　围绕以上总体要求和主要目标，今年重点做好九方面的工作：

　　（一）按照高质量发展要求推进产业强市建设

　　深入推进质量强市示范城市建设。广泛开展质量提升行动，引导企业建立健全质量管理体系，加快培育技术、标准、品牌、服务“四位一体”的质量竞争新优势。组织开展“标准化+”系列行动，鼓励和帮助企事业单位增强标准化意识和能力，积极承担和参与制修订国际标准、国家标准和行业标准。传承和弘扬无锡工匠精神、劳模精神，增强“无锡质量”“无锡品牌”理念。加强打击侵权和制售假冒伪劣商品工作，开展质量问题产品专项整治和区域集中整治，严厉查处质量违法行为。

　　深入实施现代产业发展三个“三年行动计划”。围绕行动计划，调整优化产业扶持政策，强化政策兑现，确保全面落实到位。加快推进国家传感网创新示范区建设，深入开展“互联网+”行动，推动物联网、大数据、云计算、超级计算、人工智能和实体经济深度融合，办好2018世界物联网博览会，加快推进鸿山、雪浪、慧海湾等物联网小镇和浪潮大数据产业园建设，实现物联网产业营业收入增长20%以上。积极争创“中国制造2025”国家级示范区，加快发展智能工厂、智能车间，组织推进100个智能制造重点项目，聚力打造集成电路、生物医药、新能源及新能源汽车等重点产业集群，实现规模以上工业增加值增长8%左右。加快发展生产性服务业，大力发展共享经济、数字经济、创意经济等服务业新业态、新模式，推进滨湖区省级服务业综合改革试点和梁溪区商贸流通创新发展示范区建设，推动现代服务业提质增效。

　　强化重大产业项目支撑带动作用。狠抓招商引资不放松，主攻投资体量大、科技含量高、带动能力强的产业项目，集中力量再招引一批投资超10亿美元或50亿元的重大产业项目。全力推进已签约重大项目落地开工，加快在建项目建设进度，抓好总投资超过3477亿元的市级重大产业项目建设，争取年内完成投资1050亿元。积极开展“千企技改”行动，支持企业提高产品技术、工艺装备、能效环保水平，实现全市工业投入增长8%左右。

　　大力促进乡村振兴发展。加快推进农业供给侧结构性改革，全面开展现代农业发展“343”行动，健全城乡一体化发展体制机制，促进农村一二三产业融合发展。优化农产品结构，稳定农产品供给，培育特色优势农业品牌，农业“三品”产量占比达到48%以上。强化农业物质装备和技术支撑，加强物联网技术在农业领域的推广应用，加快推进以锡山区国家现代农业产业园为龙头的百家重点园区高质量发展。鼓励多种形式适度规模经营，培育新型农业经营主体，实现省级农业龙头企业收入增长8%。完善农产品市场体系，加快苏南农副产品物流配送中心和无锡食品科技园建设。落实粮食安全责任制，提升全市粮食安全保障水平。加快推进城乡一体化示范镇、特色田园乡村建设试点，新创建一批美丽乡村休闲旅游示范村。

　　强化对实体经济的全方位服务。完善实体经济企业帮扶机制，加强土地、能源、人力资源等要素保障，进一步开展“稳心留根”活动，切实为企业发展排忧解难。加大金融服务实体经济力度，疏通金融进入实体经济的渠道，实现社会融资规模1700亿元以上，其中新增本外币贷款800亿元以上，制造业贷款继续保持正增长。增强地方国有担保公司实力，积极开展中小企业融资授信活动。大力发展产业基金、资产管理、股权投资和交易等金融新业态，引导和扶持新设产业基金规模100亿元以上。加强上市后备资源培育，新增境内外上市公司10家。

　　（二）坚持把创新作为驱动发展的第一动力

　　加强苏南国家自主创新示范区建设。落实创新驱动核心战略30条政策措施，充分发挥在锡高校和科研院所作用，推动产学研深度融合，注重开展基础研究、原始创新，积极参与国家和省重大科技计划，争取在前沿技术领域形成无锡发展核心竞争力。充分发挥无锡高新区、江阴高新区和宜兴环科园等国家高新技术园区的引领辐射作用，全面落实中关村先行先试政策，加强科技创新资源的集约利用和优化配置，推动创新成果在高新园区更多涌现。完善科研成果转化激励机制，鼓励建设区域性技术产权交易市场，扩大财政风险补偿资金对科技型中小企业的覆盖面和受益面，强化科技金融服务，促进科技成果加快转化。落实知识产权强市建设15条政策措施，强化知识产权创造、保护、运用，促进专利申请量和授权量稳定增长，万人有效发明专利拥有量达到34.5件。

　　着力发挥企业创新主体作用。落实高新技术企业所得税优惠、企业研发费用加计扣除优惠、研发投入普惠性奖励等政策，支持企业建设高水平研发机构，鼓励企业加大研发投入，实现全市企业研发经费占销售收入比重达到1.78%。实施高成长性创新型企业培育行动，加大对科技型中小企业支持力度，打造一批行业“隐形冠军”和“科技小巨人”，实现全市高新技术企业超过1800家。加强无锡军民融合公共服务平台建设，支持优势民营企业“民参军”，推动在锡军队院所“军转民”，把做大做强“两机”产业作为军民融合的重要切入点，加快推进宜兴军民融合产业园、蓝鲸军民融合创新园建设，积极争创国家级军民融合创新示范区。

　　打造科技创新创业人才高地。深入实施“太湖人才计划”升级版，搭建全方位人才服务体系，大力集聚“高精尖缺”人才智力资源，继续办好无锡（太湖）高层次人才创新创业交流大会、高层次人才交流合作洽谈会暨“百企千才高校行”等活动，引进科技创新创业领军人才（团队）企业70家。促进大众创业、万众创新与各行各业深度融合，鼓励发展创新工场、虚拟创新社区等新型孵化器，办好首届无锡市科技创新创业大赛。

　　推动形成全面创新的生动局面。积极完善有利于全面创新的政策和制度环境，全面推动产业创新、企业创新、市场创新、产品创新、业态创新和管理创新。大力营造鼓励创新、宽容失败的良好氛围，为各类市场主体开展创新活动提供有力支持，为各方面人才施展创新才能提供广阔天地。大力弘扬优秀企业家精神，完善企业家正向激励机制，形成尊重和激励企业家干事创业的社会氛围。特别要充分保护和激发民营企业家创新热情，认真贯彻国家关于产权保护的法律法规和政策措施，加快构建“亲”“清”新型政商关系，坚决破除制约民营企业发展的歧视性限制和各种隐性障碍，进一步提振民营企业家创新发展信心。

　　（三）着力推动深化改革重点任务落地见效

　　在深化供给侧结构性改革上保持定力。把“去产能”与优化产业结构结合起来，更多运用市场机制实现优胜劣汰，确保完成省下达的去产能任务。把“去库存”与促进新型城镇化结合起来，加快户籍制度改革，加大商业地产去库存力度，健全购租并举的住房制度，促进房地产市场平稳健康发展。把“去杠杆”与防范化解金融风险、政府性债务风险结合起来，强化企业自身债务杠杆约束，积极推进企业兼并重组，有效化解企业“互保链”风险，坚决打击非法集资和违法违规金融活动；积极稳妥化解地方政府性债务风险，落实好债务化解方案，严控新增债务，健全完善政府性债务管理各项制度。把“降成本”与鼓励企业挖潜增效结合起来，全面落实国家和省、市各项减税降费政策，鼓励引导企业深挖内部潜力，继续为企业降本减负200亿元以上。把“补短板”与提高发展的整体性协调性结合起来，围绕聚力创新、聚焦富民、基础设施等重点领域，补短板强弱项，提高供给质量，增强发展后劲。

　　在推进“放管服”改革上精准发力。持续推进相对集中行政许可权改革试点，健全“不见面”审批（服务）体系，全面实施并联审批、区域评估、电子审图等举措，不断巩固“3550”改革成果。着力构建适应现代化市场体系的商事制度，抓好“多证合一”“先照后证”“证照分离”和全程电子化登记等改革举措的落实和推广，推进“双随机、一公开”全覆盖，提升事中事后监管效能。完善政务服务“一张网”，提高政务服务事项在线办理比例。开展“减证便民”专项行动，构建线上线下一体化的公共服务体系。推进全市政务服务品牌化建设，进一步规范行政行为，提高政务服务效能。加快推进第二批经济发达镇行政管理体制改革和交通、建设、农业等领域的综合行政执法体制改革。深化江阴市县级集成改革试点，推进太湖新城管理体制优化调整，完成国有企事业单位公车改革任务，扎实做好承担行政职能的事业单位改革后续工作。

　　在全面落实改革举措上持续用力。积极推进财税和投融资改革，建立市（县）、区重大基础设施共建共担机制，设立城市建设创新发展基金，加快PPP项目规范运作和落地实施，开展优化税收营商环境试点。推进国有企业改革，加强国有资本资源整合，健全国有资产监督管理体制机制，完善市属企业法人治理结构，积极发展混合所有制经济。推进金融改革，积极引进和培育各类金融机构，创建物联网金融综合改革试验区，申报设立民营锡商银行和无锡金融租赁公司。推进农村土地制度和集体产权制度改革，探索“三权分置”多种实现形式，多途径发展壮大集体经济。落实供销合作社综合改革重点任务。完善统计体制，组织开展第四次全国经济普查。进一步推进医药卫生体制改革，提升医联体建设内涵，扩大家庭医生签约服务范围，加快医保支付方式改革步伐。

　　（四）推动形成开放型经济和文化旅游经济新格局

　　高水平发展开放型经济。抢抓“一带一路”发展机遇，加大外贸稳增长调结构力度，加快发展服务贸易和跨境电商，推动外贸向优质优价、优进优出转变。积极应对国际投资环境变化，放宽利用外资市场准入，拓宽利用外资渠道，强化重大外资项目招引，更大力度推动现有外资企业向地区总部、功能性总部转型升级，争取到位注册外资36亿美元。坚持“走出去”和“引进来”并重，支持帮助企业沿着“一带一路”开拓发展新空间。积极复制推广自贸区改革试点经验，做好国家级开发区赋权衔接工作，推进惠山经济开发区、江阴临港经济开发区等开发区提档升级，确保江阴综合保税区通过验收。强化开发区主阵地作用，落实考核激励机制，推动开发区主要经济指标占全市比重、在全省排位稳步提升。加快民航无锡空管站和药品进口口岸建设，申报国际邮件互换局，启动苏南硕放国际机场航空物流区和公务机基地建设前期工作，加密国际国内客运航线，探索开通洲际货运航线，增强区域性枢纽机场功能。

　　推动文化体育会展产业融合发展。健全现代文化产业体系和市场体系，加强影视传播、动漫手游、网络文化、工业设计等文化创意产业培育，加快推进无锡国家数字电影产业园二期、万达文旅城等重点项目建设，积极创建国家文化出口基地、省级文化金融合作试验区、文化金融服务中心，实现文化产业增加值占地区生产总值比重4.38%。推动体育产业创新发展，加快太湖新城国家级运动健康产业园、蠡湖水上运动基地、宜兴龙池山自行车公园、江阴海澜飞马水城等重点项目建设，提高智慧体育产业园发展水平。大力发展会展经济，积极扩大物联网、新能源、汽车、工业设计、文化艺术产业等会展的影响力和辐射效应。

　　积极创建国家全域旅游示范区。编制全域旅游发展规划，加强旅游目的地城市建设，进一步完善旅游市场体系和旅游公共服务体系。加快锡澄宜“一体两翼”旅游资源整合力度，扩大马山太湖国家旅游度假区、阳羡国家生态旅游度假区品牌效应，推进徐霞客休闲旅游度假区、宜兴国际旅游度假区、阳山生态休闲旅游度假区等重点旅游项目建设，支持惠山古镇景区创建国家5A级旅游景区。精心打造以“太湖明珠”“灵山—拈花湾”“现代影都”“运河工商文化”等为代表的特色旅游品牌，办好无锡第五届国际赏樱周和第九届无锡市国际友城交流会，实现旅游总收入增长10%。

　　（五）加快推进锡澄宜城市群协同发展

　　增强城市群协同竞争力。积极主动融入长江经济带、长三角一体化和扬子江城市群建设，同步提升无锡、江阴、宜兴城市能级，推动无锡打造长三角重要中心城市、江阴打造长江经济带重要节点城市、宜兴打造宁杭生态经济带中心城市，形成锡澄宜城市群相互依托、优势互补、协同发展、合力竞争的生动格局。深化锡澄宜产业体系分工协作，研究确定锡澄、锡宜协同发展区的空间范围、功能定位和发展策略，探索产业协作园区共建机制，鼓励和引导重大产业项目加快向江阴南部、宜兴东北部等重点协作区转移落地。

　　加快基础设施互联互通。加强无锡与江阴、宜兴城市总体规划的有机衔接，统筹谋划锡澄宜功能分区、产业分工、城镇布局、生态建设和综合交通等战略问题。加快苏锡常南部高速公路等重大基础设施建设步伐，开工建设南沿江铁路、凤翔路快速化改造、宜兴丁蜀通用机场等重点项目，基本完成锡澄城际轨道S1线一期工程前期工作，启动锡宜城际轨道S2线、宜马快速通道等项目前期工作，配合省有关部门做好盐泰锡常宜铁路、锡太高速、锡宜高速改扩建等项目前期工作。

　　推进公共服务共建共享。建立开放的就业服务信息平台，加快实现社会保险关系无障碍转移。完善市域客运公交网络，探索开通城际公交线路，推进公交资费同城化。落实基本公共服务功能配置标准，整合区域教育、医疗、养老等资源，促进教育均衡协调发展，推动医疗卫生、养老服务共享。统一区域环境准入标准，加强环境质量联合监测。深化文化体育外事交流，合作举办群众性文化演出和体育比赛，共同申办全国性、国际性重大文化活动和体育赛事。

　　（六）提高保障和改善民生水平

　　持续促进富民增收。坚持就业优先战略和积极就业政策，开展职业技能培训，促进高校毕业生等重点群体就业，实现城镇新增就业11万人。鼓励创业带动就业，扶持自主创业1万人。完善职工工资正常增长机制，拓宽居民劳动收入和财产性收入渠道，加快农民合作经济组织体系建设，履行好政府再分配调节职能，做到在经济增长的同时实现居民收入同步增长、在劳动生产率提高的同时实现劳动报酬同步提高，进一步缩小城乡居民收入差距。

　　健全兜底保障体系。推动社会保障从制度全覆盖向人群全覆盖转变，实现企业职工养老、医疗、失业、工伤和生育保险分别扩面5万、5万、3万、3万和3万人以上，探索推广江阴村级医疗互助制度。大力推进“阳光扶贫”，实施低收入群体和经济薄弱村脱困致富工程，健全最低生活保障标准自然增长机制，加大因病因残致贫家庭救助力度，强化住房保障、社会救助、社会福利、慈善事业、优抚安置等政策的托底保障功能。完善居家养老服务机制，探索实施长期护理保险制度，积极推进中民养老服务中心等项目建设。

　　加快教育现代化步伐。全面提升各级各类教育发展水平，加大幼儿园、中小学布局优化和建设力度，推进义务教育学校标准化建设，努力解决基础教育面临的学生课外负担重、“择校热”等问题。实施普通高中办学品质提升计划。加大职业教育校企合作、产教融合力度，深化现代学徒制试点，推动开放大学创新发展。支持江南大学建设世界一流学科和高水平医学院，做好太湖学院“校中村”征收拆迁、支持学院通过教育部本科教学水平评估，实现南京信息工程大学滨江学院无锡校区入驻新生，做好江南影视艺术职业学院“专升本”工作。推进创办中外合作大学，巩固提升与东南大学等高校合作共建成果。

　　深化“健康无锡”建设。落实“健康无锡2030”规划纲要，争创全国健康城市示范市。增加优质医疗资源供给，加快推进地方医疗机构设施建设，完成第四人民医院新院区主体工程，加强基层医疗卫生服务体系和全科医生队伍建设。实施“科教强卫”工程，加大医疗卫生人才引育和重点专、学科建设力度。坚持中西医并重，传承发展中医药事业。坚持预防为主方针，强化慢性病防控，不断提升公共卫生服务效能。促进生育政策和相关经济社会政策配套衔接，进一步加强妇幼健康服务能力建设。深入实施食品安全战略，建立健全食品溯源体系，创建国家食品安全示范城市。

　　繁荣文化体育事业。扎实开展书香城市创建，推进市文化馆、美术馆、基层综合性文化服务中心等公共文化设施建设。鼓励创作具有地方特色的文艺作品，推动锡剧艺术繁荣发展。加强历史文化遗产保护和合理利用，加快推进惠山古镇二期建设和申遗工作。提升公共体育服务水平，推动大型体育场馆免费或低收费开放，争创全国体育旅游示范城市和全国运动健康城市。积极备战第十九届省运会，办好2018年世界击剑锦标赛等一批重大国际体育赛事。

　　（七）加强城乡建设管理和社会治理

　　提高城乡建设和管理水平。坚持规划引领，进一步做好城市总体规划修编、镇村规划编制工作，推进城市设计和历史城区“双修”试点。突出产城融合，支持引导新城开发和特色小镇健康发展，推动太湖新城、锡东新城、惠山新城、蠡湖新城和太湖影视小镇等特色小镇加快产业导入、完善配套服务。加快实施棚户区、旧住宅区更新改造，完善老新村改造长效管理机制。加强老城区环境卫生整治，有效解决背街小巷“脏乱差”问题。推进城市管理执法体制改革，完成数字城管二期升级工程，不断提高城市管理的网格化、数字化水平。进一步加强农村居民住房建设管理，积极推进“厕所革命”，建立健全村庄综合整治长效管理机制。加快建设建筑垃圾终端处置消纳场所，推动建筑垃圾分类处置和资源化利用，严肃查处各类偷倒行为。坚决遏制新增违法建设，逐步清理和整治存量违法建设。

　　加强城乡基础设施建设。加快推进轨道交通建设，地铁3号线一期实现全线隧道贯通，地铁4号线一期进入盾构施工阶段。加快钱皋路、环山东路、新锡路北延等项目建设，力争年内建成通车。推动公共交通优先发展，优化调整公交线路，开通更多“定制公交”“旅游公交”，促进公共自行车、共享单车和网约车规范有序发展。加强城市地下综合管网和海绵城市建设管理。加快实施新孟河拓浚延伸工程、望虞河西岸控制工程、锡澄运河北排扩大工程、白屈港整治工程，完成新沟河拓浚延伸工程。实施新型智慧城市建设行动计划，打造智慧城市建设先行示范区。

　　创新和加强社会治理。深化政社互动，推广“三社联动”经验，推进网格化社会治理，进一步完善社区减负长效机制，加强对特殊人群的心理健康服务，推动形成共建共治共享的社会治理格局。推进社会信用体系建设，倡导健康文明生活方式，巩固提升全国文明城市群创建成果。完善落实拥军政策，增强军人荣誉感。健全安全生产责任体系，完成全国安全生产领域改革发展试点任务，加强基层安全生产监管队伍建设，深化安全生产隐患排查治理、重点行业领域专项整治，坚决防范发生重特大安全事故，确保全市安全生产形势持续向好。组织开展信访突出问题大化解大突破专项行动，完善社会矛盾纠纷多元化解机制。深入推进平安无锡和“雪亮工程”全国示范城市建设，大力推进智慧警务建设，健全立体化信息化社会治安防控体系，严厉打击各类违法犯罪行为。

　　（八）强化环境整治和生态保护

　　把太湖治理放在重中之重位置。落实太湖治理和河道综合整治“1+4”方案，加快推进重点治太工程，强化应急防控措施，确保饮用水安全、确保不发生大面积湖泛。深入推进工业污染、生活污染和农业面源污染防治，加快规模养殖场改造提升，完成排水达标区创建118块以上。严格落实“河长制”“断面长制”监管责任，突出控源截污和水系连通两大关键举措，全面推进161条河道和38条黑臭水体整治，打通一批“断头浜”，促进形成河道环流系统，确保45个国家、省考核断面水质稳定达标。

　　着力整治突出环境问题。抓好大气污染综合治理，切实加强挥发性有机物治理、机动车船污染防治、扬尘整治等重点工作，实现PM2.5平均浓度持续下降、城市空气质量优良天数比例持续上升。强化土壤环境综合监管，抓好重点区域污染土壤综合治理与修复试点示范。加快固体废弃物处置设施建设，提高锡东垃圾焚烧发电厂运行水平，开工建设惠联餐厨垃圾处理厂、惠山飞灰填埋场、新吴工业固废焚烧处理厂。积极推进生活垃圾分类工作，实现生活垃圾分类设施覆盖率达40%。继续开展“绿刃2018”环保专项行动，清理整顿重污染小型企业，严厉打击各类环境违法行为。

　　深入推进绿色低碳发展。严格执行主体功能区战略，科学划定城市开发边界、生态保护红线和永久性基本农田保护线。加快推进太湖生态保护圈、长江生态安全带、宜兴生态保护引领区和中瑞低碳生态城建设，努力形成典型示范。扎实开展能源消耗总量和强度“双控”行动，整治燃煤工业窑炉34座，确保完成省下达的减煤任务。加快化工行业结构调整，关停化工企业154家，完成化工企业转型升级280家。开展工业企业资源利用绩效管理，促进低端产业和低效企业加快转型。积极推进企业循环式生产、产业循环式组合、园区循环式改造，提高全社会资源产出率和综合利用水平。发挥公共机构节能管理示范作用，引导全社会形成绿色生产生活方式。

　　完善生态文明体制机制。严格落实环境保护“党政同责”和“一岗双责”，规范开展领导干部自然资源资产离任审计，深入推进环保机构监测监察执法垂直管理制度改革。落实生态补偿机制，实施与污染物排放总量挂钩的财政政策，积极开展排污许可证核发工作。推进碳排放权交易市场建设，加强环境风险评估工作。完善环境污染责任保险制度。建立健全污染源档案和信息数据库，做好第二次全国污染源普查工作。

　　（九）努力建设担当高效廉洁政府

　　坚持依法行政。落实《无锡市法治政府建设规划》，提升政府立法质量，完善法律顾问制度，加强政府规范性文件的备案审查，推进重大行政决策目录管理，促进政府决策民主化、法治化、科学化。加大行政执法监督力度，规范自由裁量权行使，促进严格规范公正文明执法。依法接受市人大及其常委会的监督，积极支持市政协履行政治协商、民主监督和参政议政职能，提高人大代表建议和政协委员提案的办理质量。推进司法行政“智慧法务”平台建设，做好普法宣传工作，增强全民法治观念。

　　强化责任担当。大力倡导保持锐意进取的勇气、敢为人先的锐气、蓬勃向上的朝气，弘扬“定了干、马上办、办到底”的作风。进一步增强群众观念和群众感情，真心实意为群众办实事、解难事，主动当好服务群众和企业的“店小二”。完善激励机制和容错纠错机制，旗帜鲜明为敢于担当、踏实做事、不谋私利的干部撑腰鼓劲，坚决整治工作落实不力、效能低下、敷衍塞责等“庸懒散”行为，推动形成干事创业、争先创优的良好氛围。

　　认真落实全面从严治党主体责任和“一岗双责”。推动和督促全市政府系统各级党组（党委）切实履行主体责任，领导班子成员切实履行“一岗双责”，做到把党风廉政建设与经济社会发展工作同部署、同落实、同检查、同考核。认真贯彻执行中央八项规定《实施细则》及省、市委贯彻落实的《具体办法》，坚决防止“四风”问题反弹回潮。加强审计监督，落实审计发现问题整改“销号”制度。坚持把纪律挺在前面，用好监督执纪“四种形态”，突出抓早抓小。加大违纪违法案件查处力度，坚决纠正损害群众利益的不正之风。

　　各位代表，保障和改善民生是政府工作的根本出发点和落脚点。今年我们将继续加大投入力度，着力办好民生10件实事：一是环境污染防治项目。实施挥发性有机物治理重点工程项目150个。重点实施西新河、梅东河等24条黑臭河道综合整治。加快建设市区工业固体（危险）废物安全填埋二期工程。二是扶贫脱困和社会公益扶助项目。实现全市低保家庭人均年收入达到10000元以上，脱困转化经济薄弱村26个，“阳光扶贫”覆盖2万余户、3万余人。建立市区特定困难对象医疗救助责任保险制度，覆盖5万余人次重大病患者。1100家居家养老服务机构全面实施综合责任保险。全市建成135个“残疾人之家”。开展公益性应急救护培训，培训初级救护员7000人，普及培训57000人。三是重点交通基础设施建设项目。完成江海西路和蠡湖大道快速化改造工程，实施凤翔路快速化改造项目。加快跨区道路建设，开工建设340省道、飞凤路、锡州路一期等项目。加快区域交通改善道路建设，完成平湖路（清源路—具区路）工程，开工建设渔港路、新惠路、惠峰路、运河东路大修等项目。四是城市公共交通建设项目。新辟和优化调整10条公交线路，新增、更新新能源和低碳公交车150辆。投放市民卡综合自助办理终端设备500台，建设无锡地铁“便民云服务平台”。五是市政配套设施改造项目。完成老旧自来水管网改造50公里。完成自来水水表出户、抄表到户3000户。完成锡澄水厂深度处理主体工程。新增天然气用户5万户，扩大清洁能源使用范围。六是农贸市场改造提升项目。改造提升市区农贸市场8家。打造农贸市场综合管理平台，推进全市农贸市场远程智慧监管。启动建设市食品安全检验检测中心。七是旧住宅区综合整治改造和平安无锡建设项目。完成旧住宅区整治改造220万平方米，棚户区（危旧房、城中村）改造50万平方米。同步完成城区老旧小区技防设施建设。市区新增城市绿地面积200万平方米。完成市区15座环卫公厕改造。实施“雪亮工程”建设项目，实现社会面公共安全视频监控联网应用。八是智慧城市建设项目。启动“智慧食安”信息化建设项目，打造全市食品药品安全智慧监管和风险防控信息平台。建设无锡市电梯安全监管物联网系统，打造“智慧电梯”。启动实施物联网消防装备智能管理系统，打造“智慧消防”。完成全市范围内50家快递末端网点信息监管平台建设，强化快递监管。打造“无锡通”综合信息服务平台，建设4G无锡无线网工程，建成一批千兆宽带示范小区。实施物联网智能停车场项目，建设智慧家庭和智能家庭安全守护基础网。九是文化体育卫生惠民项目。建设50个道德教育实践场所。培育提升300支特色文化团队。完成150场“文艺进万家”活动。升级改造新体中心体育馆，建设智慧体育场馆。第四人民医院新院区完成建设。新锡山人民医院、新瑞医院建成投用。10家基层医疗机构完成提档升级改造。在火车站、中央汽车站、机场、重点景区等大型公共场所有计划配置自动体外除颤仪（AED），完善应急救护网络。十是教育事业建设项目。新建、改扩建义务教育学校项目17个、幼儿园项目7个，续建义务教育学校项目26个、幼儿园项目16个。南京信息工程大学滨江学院无锡校区一期工程完工投用，正式招生。组建市级教师发展学院。

　　各位代表，蓝图已经绘就，航向已经标明，号角已经吹响。让我们紧密地团结在以习近平同志为核心的党中央周围，在中共无锡市委的坚强领导下，紧紧依靠全市人民，凝心聚力、开拓进取，以永不懈怠的精神状态和一往无前的奋斗姿态，在高水平全面建成小康社会、加快建设“强富美高”新无锡的征程上阔步前进，为谱写新时代中国特色社会主义无锡实践的新篇章而努力奋斗！
